# Parental preferences for sex of children in Nigeria: Cultural influences and family structure

**DOI:** 10.1371/journal.pone.0327474

**Published:** 2025-07-11

**Authors:** Alex Bawuah, Michael Sarfo, Godness Kye Biney, Francis Appiah, Linus Baatiema, Sanni Yaya

**Affiliations:** 1 School of Global Studies, Faculty of Social Science, University of Sussex, Brighton, United Kingdom; 2 School of Human and Health Sciences, University of Huddersfield, United Kingdom; 3 Department of Biostatistics and Epidemiology, School of Public Health and Health Sciences, University of Massachusetts Amherst, Amherst, Massachusetts, United States of America; 4 School of Graduate Studies, Lingnan University, Hong Kong SAR, China; 5 Department of Population, Family and Reproductive Health, School of Public Health, Kwame Nkrumah University of Science and Technology, Kumasi, Ghana; 6 Ghana Health Service, Upper West Regional Health Directorate, Wa, Ghana; 7 L&E Research Consult Ltd, Wa, Upper West Region, Ghana; 8 Centre for Migration, Security and International Relations, Faculty of Policy, University of Business and Integrated Development Studies; 9 George Institute for Global Health, Imperial College London, London, United Kingdom; Federal University of Agriculture Abeokuta, NIGERIA

## Abstract

**Background:**

Nigeria is characterized by deeply rooted traditional practices that often amplify gender bias. Despite this, there is limited research examining the relationship between cultural and family characteristics and parental sex preference in Nigeria. To address this gap, we investigated how cultural factors and family structure is associated with parental sex preferences in the country.

**Methods:**

This study utilized data from the 2018 Nigeria Demographic and Health Surveys (NDHS). Parental sex preference for children served as the outcome variable, while family structure and cultural background were the explanatory variables. Descriptive analyses, including frequencies, percentages, and cross-tabulations, were used to characterize the sample. Due to the unordered categorical nature of the outcome variable, a multinomial logistic regression model was employed to assess the impact of culture and family structure on parental sex preference.

**Results:**

A higher proportion of women preferred more girls compared to men (15.64% vs 6.85%), while more men expressed a preference for boys than women (52.48% vs 26.2%). Men with more sons were significantly more likely (RRR = 1.48, 95% CI = 1.23–1.78) to prefer more boys over an equal number of boys and girls, compared to men with equal numbers of sons and daughters. Similarly, women with more sons were more likely (RRR = 1.60, 95% CI = 1.45–1.77) to prefer more boys rather than an equal number of boys and girls, compared to women with equal numbers of sons and daughters.

**Conclusion:**

These findings shed light for understanding gender dynamics and informing policies that promote gender equality and balanced family structures. Such policies are critical for enhancing family planning practices and advancing the Sustainable Development Goals (SDGs) by 2030. With only six years left to meet these targets, we urge all stakeholders to collaborate and intensify efforts to drive meaningful progress.

## Introduction

With nearly six years to end the Sustainable Development Goals, it is imperative to realise that issues of gender inequality, from a family perspective through to the national standpoint, continue to linger prominently in low- and middle-income countries and are deeply rooted in societies globally [1,2]. Sex preferences, while often discussed in relation to gender inequality, are not necessarily indicative of discriminatory treatment within households. Instead, they should be understood as reflections of broader societal norms, cultural traditions, and psychological influences. Parents may express preferences for a specific sex without engaging in unequal treatment of their children. This study, therefore, adopts a socio-cultural and psychological lens to better analyze these preferences beyond policy-oriented interpretations [3].

Parental sex preferences remain a significant aspect of reproductive decision-making in Nigeria, influenced by cultural, religious, and socioeconomic factors. Theoretical models, such as Bongaarts’ fertility and gender preference framework and Becker’s Theory of Fertility Preferences, help explain how economic and demographic factors interact with cultural norms to shape these preferences. These models provide a structured approach to understanding the underlying motivations for parental preferences and their demographic implications.

According to the United Nations Office of Human Rights report in 2017, Nigeria has adopted a policy and legal framework to help tackle the growing menace of gender bias, specifically against women [4]. The sex ratio at birth is primarily determined by biological factors, with a natural range of approximately 103–107 male births per 100 female births globally. In Nigeria, the observed sex ratio at birth (106:100) falls within this expected biological norm, suggesting that parental sex preferences do not significantly alter birth outcomes. Unlike in some Asian countries where sex-selective practices influence demographic trends, Nigeria does not exhibit strong evidence of such practices. Studies, including those published in the African Journal of Reproductive Health (26:4), affirm that variations in sex ratio at birth in sub-Saharan Africa are not driven by sex-selective abortion or parental behaviours [5]

According to a study conducted by Odusina et al. [6], Nigerian women have the tendency to continue to give birth until they have achieved their desired number of sons, a practice that has skyrocketed the country’s birth rate. This assertion is supported by a World Bank report in 2021 which estimates that Nigeria’s total fertility rate was 5.2 per woman, which is one of the highest in the world [7]. Nigeria is one of the countries with deeply rooted traditional practices that tend to exacerbate gender bias [8–10]. Relatedly, educational disparities are stark, with girls from the northern part of the country particularly disadvantaged, as societal and cultural norms prioritise the education of boys [11,12] Similarly, Nigerian women experience profound biases in healthcare, especially in reproductive rights, where societal expectations and male-dominated decision-making severely limit their access to family planning and contribute to high maternal mortality rates [13–15]. These biases extend to political representation, where women are starkly underrepresented, reflecting the deep-rooted patriarchal norms that continue to shape societal structures

Due to the cultural orientation and inheritance practices in Nigeria, women are usually under intense pressure to birth a son for their husbands and consolidate their marriage [16]. Consequently, women who give birth to only daughters are usually unhappy and stressed, facing disappointment from their husbands and family [10]. This phenomenon has led to some men requesting early ultrasonography to determine the sex of the foetus, resulting in low birth spacing and episodes of pregnancy terminations when the desired sex is not achieved [17–19]

It is asserted that Nigerian women generally accept larger family sizes to achieve sex balance [8,10]. This trend is evident in national statistics, showing that Nigerian women desire large families, with currently married women wanting an average of 7.1 children and all women averaging 6.5 children [20]. Only 9% of Nigerian women consider three or fewer children ideal [20]. Nigeria’s population is estimated to increase by 6,031,859 people and reach 232,198,302 by the beginning of 2025 [21]. This increase is expected to be driven by natural population growth, where the number of births will surpass the number of deaths by approximately 6,111,017 [21]. This is further underpinned by the 2024 global sex ratio report which estimates the sex ratio in Nigeria as 1.0263, higher than the global sex ratio of 100.978 males per 100 females [22].

Given the complexities and persistence of sex preference even in the wake of modernity, it is imperative to measure contextually relevant variables such as culture and family orientation and their association with sex preference in Nigeria, a country that is multicultural and religiously inclined. To this end, our study examines parental sex preferences in Nigeria using established demographic and economic theories that explain fertility decisions and gender preferences. Specifically, Bongaarts’ fertility and gender preference model provides a framework for analyzing how demographic factors, such as parity progression and fertility regulation, influence parental preferences for sons or daughters [23]. Similarly, Becker’s Theory of Fertility Preferences suggests that economic and utility-based considerations play a role in shaping parental choices, particularly in contexts where male children are perceived as having greater economic or social advantages [24].

Based on these established demographic and economic theories, we formulate the following hypotheses to guide our analysis: (1) Parents with more sons are less likely to express a preference for additional boys compared to those with more daughters; (2) Ethnic and religious differences significantly influence sex preferences, with northern Nigerian groups more likely to prefer boys due to patrilineal inheritance norms; and (3) Socioeconomic factors, such as maternal education and employment status, are associated with a reduced preference for male children. These hypotheses are tested using multinomial logistic regression models.

## Methods

### Data source

The study used data from the 2018 Nigeria Demographic and Health Surveys (NDHS). The 2018 NDHS is a nationally representative survey that provides up-to-date information on demographic and health indicators including fertility, family planning, maternal and child health, adult and childhood mortality, women’s empowerment, domestic violence, malaria, HIV/AIDS and other sexually transmitted infections (STIs), and other health-related issues [25].

The NDHS employed a two-stage sampling process. In the first stage, 1,400 enumeration areas (EAs) were selected with probability proportional to EA size. A household listing operation was carried out in all selected EAs, and the resulting lists of households served as a sampling frame for the selection of households in the second stage [25]. In the second stage, a fixed number of 30 households were selected in every cluster through equal probability systematic sampling, resulting in a total sample size of approximately 40,427 households. Interviews were then conducted only in the selected households [25].

The sample for this study includes men and women who were interviewed in the selected households. Overall, 41,821 women aged 15–49 years, and 13,311 men aged 15–59 were successfully interviewed [25]. After cleaning the data and dropping all missing observations (for this study). Observations with missing data on primary independent or dependent variables were removed using listwise deletion. This approach ensures consistency across all model estimates. After the deletion, we ended up with a sample size of 43,398 which is made up of 33,008 women and 10,390 men.

Based on established demographic and economic models, the following hypotheses are tested in this study:

H1: Parents with more sons are less likely to express a preference for additional boys compared to those with more daughters.

H2: Ethnic and religious differences significantly influence sex preferences, with northern Nigerian groups more likely to prefer boys due to patrilineal inheritance norms.

H3: Socioeconomic factors, such as maternal education and employment status, are associated with a reduced preference for male children.

### Variables

Parental sex preference of children was the outcome variable in the study. The DHS data provided information about the sex preferences of children for the men and women who were interviewed. The precise question asked is “If you could go back in time, you did not have any children and could choose exactly the number of children to have in your whole life, how many would that be? How many boys? How many girls”. We thus classified the responses into three: 1 = prefer an equal number of boys and girls (B = G); 2 = prefer more boys (B > G); 3 = prefer more girls (G > B).

Two explanatory variables were used in the study: the respondent’s family structure and culture. In the DHS survey, the respondents were asked about the number of sons and daughters they have and how many of them are alive. We thus define family structure as the number of sons and daughters who are alive among the children born to the respondent. We categorise family structure into four: 1 = has the same number of sons and daughters (S = D); 2 = has more sons than daughters (S > D), 3 = has more daughters than sons (D > S); 4 = has no children.

Ethnicity was used as a proxy for culture. Ethnicity refers to a type of social identity based on cultural background, shared lifestyles and shared experiences [26]. It encapsulates long-standing traditions, norms, and values that influence family structures and reproductive behaviours. Prior studies have established that ethnic identity is strongly correlated with cultural beliefs, particularly in contexts where social norms are transmitted across generations within ethnic groups [26]. However, we acknowledge that culture is multidimensional and may also be shaped by urbanization, education, and religious affiliation. To account for this, we controlled for education and religion in the regression models to examine whether these factors independently influence parental sex preferences.

We further account for the following variables: age, education, residence, wealth, marital status, employment status, frequency of listening to radio, frequency of watching television, and administrative region. These covariates were selected based on their significant correlation with the outcome variable [26] as well as their availability in the DHS dataset. We maintained the coding for all the covariates as found in the DHS dataset.

### Data analysis

The data were analysed with STATA version 16. Frequency, percentages, and cross-tabulation were used to describe the sample. Given the unordered categorical nature of the outcome variable, we employed a multinomial logistic regression (MLR) model to examine how culture and family structure is associated with parental sex preference [27]. While sex preference categories (prefer more boys, prefer equal number of boys and girls, prefer more girls) may appear to have a natural ranking, they are conceptually distinct and do not follow a strictly ordinal pattern. Ordered logit regression assumes that the difference between preferring more boys versus preferring an equal number of boys and girls is the same as the difference between preferring an equal number of boys and girls versus preferring more girls. However, this assumption does not hold in our case, as parental motivations for each preference category are likely driven by different factors rather than existing along a linear continuum. Additionally, preliminary tests using ordered logit models produced results that were less interpretable and did not capture the nuances in parental sex preferences as effectively as MLR. Therefore, we rely on MLR, which does not impose restrictive assumptions about the ordering of outcome categories, allowing for a more flexible and accurate analysis of sex preferences.

Prior to the regression analysis, we conducted a multicollinearity diagnostic test using the Variance Inflation Factor (VIF) to determine potential collinearity between the variables. The results showed moderate correlation levels but did not exceed the conventional VIF threshold (5), indicating that multicollinearity is not a major concern. The VIF results are provided in Table A1 in S1 File.

Bivariate regression analysis was initially conducted to estimate the unadjusted relative risk ratios (RRR) for each determinant (Tables A2 and A3). Afterwards, a multivariable regression was conducted to estimate the adjusted relative risk ratios (RRR) for all determinants.

### Ethical consideration

We used a secondary dataset that is freely available to the public from the DHS Programme, therefore no ethical approval was requested. The dataset is anonymised and more details regarding its ethical standards are available at http://goo.gl/ny8T6X.

## Results

### Descriptive statistics of the samples

**Fig 1:**
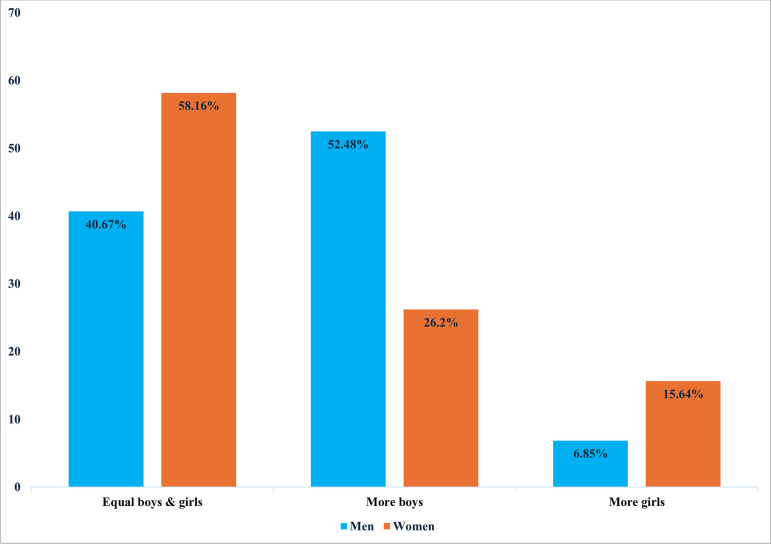
Distribution of sex preferences among men and women, Nigeria, 2018 DHS.

Fig 1 shows that the proportion of women who prefer an equal number of boys and girls is more than the proportion of men who prefer an equal number of boys and girls (58.16% vs 40.67). Similarly, the proportion of women who prefer more girls is more than the proportion of men who prefer more girls (15.64% vs 6.85%). However, the proportion of men who prefer more boys is more than the proportion of women who prefer more boys (52.48% vs 26.2%). Thus, whereas men prefer more boys, women prefer an equal number of boys and girls. Furthermore, both men and women prefer less girls.

Table 1 presents the results of the descriptive summary of the study’s sample. It shows that for both men and women, a higher proportion of them have no children (men = 41.73%, women = 31.09%). Also, a higher proportion of the men and women are Hausa (men = 24.7%, women = 23.89%). Furthermore, a higher proportion of the men and women are aged between 15–19 (men = 18.33%, women = 21.11%), respondents have secondary education (men = 48.91%, women = 42.84%), live in rural areas (men = 57.09%, women = 58.03%), in the richer wealth category (men = 22.71%, women = 22.10%), married (men = 58.25%, women = 64.49%), and employed (men = 86.45%, women = 65.33%), listens to radio at least once a week (men = 41.52%, women = 30.92%), and do not watch television (men = 38.61%, women = 46.48%).

**Table 1:** Descriptive statistics of the sample, Nigeria 2018 DHS.

	Men = 10,390		Women = 33,008	
	Frequency	%	Frequency	%
**Family structure**				
Equal sons & daughters	1,150	11.07	4,327	13.11
Sons > daughters	2,600	25.02	9,535	28.89
Daughters > sons	2,304	22.18	8,884	26.91
No children	4,336	41.73	10,262	31.09
**Ethnicity**				
Ekoi			268	0.81
Fulani	548	5.27	2,052	6.22
Hausa	2,566	24.7	7,885	23.89
Ibibio	202	1.94	757	2.29
Igala	140	1.35	340	1.03
Igbo	1,898	18.27	6,217	18.83
Ijaw/Izon	339	3.26	1,109	3.36
Kanuri/Beriberi	149	1.43	569	1.72
Tiv	200	1.92	940	2.85
Yoruba	1,553	14.95	4,062	12.31
Other	2,795	26.9	8,809	26.69
**Age**				
15-19	1,905	18.33	6,967	21.11
20-24	1,230	11.84	5,534	16.77
25-29	1,281	12.33	5,717	17.32
30-34	1,358	13.07	4,683	14.19
35-39	1,416	13.63	4,199	12.72
40-44	1,196	11.51	3,053	9.25
45-49	901	8.67	2,855	8.65
50-54	639	6.15		
55-59	464	4.47		
**Education level**				
No education	1,998	19.23	10,277	31.13
Primary	1,522	14.65	4,912	14.88
Secondary	5,082	48.91	14,142	42.84
Higher	1,788	17.21	3,677	11.14
**Residence**				
Urban	4,458	42.91	13,855	41.97
Rural	5,932	57.09	19,153	58.03
**Religion**				
Christian	5,565	53.56	17,595	53.31
Islam	4,726	45.49	15,089	45.71
Traditionalist/Other	99	0.95	324	0.98
**Wealth**				
Poorest	1,708	16.44	5,672	17.18
Poorer	1,853	17.83	6,245	18.92
Middle	2,266	21.81	7,031	21.30
Richer	2,360	22.71	7,294	22.10
Richest	2,203	21.20	6,766	20.50
**Marital status**				
Never in union	3,987	38.37	9,118	27.62
Married	6,052	58.25	21,286	64.49
Living with partner	197	1.90	801	2.43
Widowed	49	0.47	904	2.74
Divorced	56	0.54	413	1.25
Separated	49	0.47	486	1.47
**Employment status**				
Unemployed	1,408	13.55	11,445	34.67
Employed	8,982	86.45	21,563	65.33
**Frequency of listening to radio**				
Not at all	3,260	31.38	14,029	42.5
Less than once a week	2,816	27.1	8,774	26.58
At least once a week	4,314	41.52	10,205	30.92
**Frequency of watching television**				
Not at all	4,012	38.61	15,342	46.48
Less than once a week	2,861	27.54	6,778	20.53
At least once a week	3,517	33.85	10,888	32.99
**Region**				
North central	1,769	17.03	6,147	18.62
North east	1,654	15.92	5,269	15.96
North west	2,131	20.51	7,521	22.79
South east	1,593	15.33	5,252	15.91
South south	1,510	14.53	4,544	13.77
South west	1,733	16.68	4,275	12.95

### The relationship between family structure and parental sex preferences among men and women in Nigeria

Table 2 shows the results (MLR) of the relationship between family structure and parental sex preferences among men and women in Nigeria. The model’s log pseudolikelihood value (men sample = −8,944,000,000, women sample = −30,330,000,000) suggests a good overall fit, indicating that the independent variables effectively capture the variation in sex preferences. Additionally, the significance of the Wald χ² test statistic of value (men sample = 310.63, women sample = 692.03) confirms that at least one predictor variable significantly influences the outcome categories, reinforcing the model’s explanatory power.

**Table 2:** Relationship between family structure and parental sex preferences among men and women in Nigeria.

	Men 15–59		Women 15–49	
	Ref; B = G	Ref; B = G	Ref; B = G	Ref; B = G
	B > G	G > B	B > G	G > B
	RRR	RRR	RRR	RRR
Variables	(95%CI)	(95%CI)	(95%CI)	(95%CI)
**Family composition (Ref; Sons = Daughters)**				
Sons > daughters	1.47***	1.00	1.60***	1.15*
	(1.22 - 1.78)	(0.70 - 1.44)	(1.45 - 1.77)	(1.01 - 1.31)
Daughters > sons	1.03	2.08***	1.02	1.65***
	(0.87 - 1.22)	(1.52 - 2.84)	(0.92 - 1.13)	(1.41 - 1.92)
No sons & daughters	1.14	1.40	1.35***	1.43***
	(0.86 - 1.51)	(0.81 - 2.42)	(1.18 - 1.55)	(1.19 - 1.71)
**Age (Ref; 15–19)**				
20-24	1.08	0.89	1.06	1.04
	(0.89 - 1.31)	(0.62 - 1.27)	(0.95 - 1.19)	(0.92 - 1.18)
25-29	1.02	0.96	1.05	1.04
	(0.82 - 1.26)	(0.65 - 1.41)	(0.92 - 1.19)	(0.90 - 1.20)
30-34	1.15	1.02	0.99	0.90
	(0.90 - 1.46)	(0.63 - 1.64)	(0.87 - 1.13)	(0.77 - 1.06)
35-39	1.07	1.38	0.98	1.07
	(0.83 - 1.39)	(0.83 - 2.31)	(0.85 - 1.13)	(0.89 - 1.29)
40-44	1.05	1.25	0.90	0.99
	(0.81 - 1.35)	(0.75 - 2.08)	(0.77 - 1.05)	(0.82 - 1.18)
45-49	1.21	1.08	0.91	1.01
	(0.91 - 1.62)	(0.62 - 1.89)	(0.78 - 1.06)	(0.84 - 1.21)
50-54	0.99	1.19		
	(0.72 - 1.35)	(0.67 - 2.12)		
55-59	1.00	0.70		
	(0.69 - 1.44)	(0.36 - 1.35)		
**Education (Ref; None)**				
Primary	0.96	0.98	1.23***	1.00
	(0.80 - 1.14)	(0.68 - 1.41)	(1.10 - 1.38)	(0.88 - 1.15)
Secondary	0.94	1.14	1.17*	0.99
	(0.79 - 1.11)	(0.82 - 1.58)	(1.03 - 1.33)	(0.85 - 1.15)
Higher	1.00	1.28	1.27**	1.03
	(0.81 - 1.23)	(0.86 - 1.90)	(1.08 - 1.49)	(0.84 - 1.27)
**Residence (Ref; Urban)**				
Rural	0.88	0.80	0.88**	1.01
	(0.77 - 1.01)	(0.63 - 1.01)	(0.79 - 0.97)	(0.89 - 1.13)
**Religion (Ref; Christian)**				
Islam	1.26**	0.97	1.06	1.31**
	(1.08 - 1.47)	(0.71 - 1.32)	(0.94 - 1.19)	(1.11 - 1.55)
Traditionalist/Other	1.30	1.47	1.28	1.05
	(0.80 - 2.13)	(0.59 - 3.66)	(0.84 - 1.95)	(0.72 - 1.54)
**Wealth (Ref; Poorest)**				
Poorer	1.03	0.99	0.86**	0.87
	(0.84 - 1.25)	(0.71 - 1.36)	(0.77 - 0.96)	(0.76 - 1.00)
Middle	1.02	0.93	0.81**	0.95
	(0.84 - 1.24)	(0.65 - 1.32)	(0.70 - 0.93)	(0.81 - 1.11)
Richer	0.88	0.99	0.77***	1.02
	(0.71 - 1.09)	(0.66 - 1.49)	(0.66 - 0.89)	(0.86 - 1.21)
Richest	0.71**	0.71	0.75***	0.94
	(0.56 - 0.90)	(0.45 - 1.13)	(0.64 - 0.87)	(0.79 - 1.13)
**Marital status (Ref; Never in union)**				
Married	0.89	0.90	1.12	0.88
	(0.69 - 1.14)	(0.53 - 1.53)	(0.98 - 1.29)	(0.76 - 1.03)
Living with partner	1.03	1.04	1.33*	0.96
	(0.60 - 1.75)	(0.48 - 2.28)	(1.04 - 1.70)	(0.75 - 1.23)
Widowed	0.73	0.72	1.30*	0.91
	(0.34 - 1.58)	(0.17 - 3.07)	(1.04 - 1.62)	(0.69 - 1.20)
Divorced	0.79	0.91	0.89	0.90
	(0.41 - 1.54)	(0.23 - 3.63)	(0.67 - 1.17)	(0.62 - 1.31)
Separated	0.98	1.52	1.22	0.98
	(0.48 - 2.03)	(0.50 - 4.59)	(0.93 - 1.59)	(0.72 - 1.36)
**Employment status (Ref; Unemployed)**				
Employed	1.03	1.06	1.08*	0.96
	(0.87 - 1.22)	(0.76 - 1.49)	(1.00 - 1.17)	(0.88 - 1.05)
**Frequency of listening to radio (Ref; Not at all)**				
Less than once a week	0.89	0.69*	0.85***	0.97
	(0.76 - 1.06)	(0.52 - 0.93)	(0.77 - 0.92)	(0.87 - 1.07)
At least once a week	0.91	0.77	0.81***	0.82***
	(0.77 - 1.08)	(0.57 - 1.05)	(0.75 - 0.89)	(0.74 - 0.92)
**Frequency of watching television (Ref; Not at all)**				
Less than once a week	1.00	0.91	1.04	1.02
	(0.85 - 1.19)	(0.68 - 1.21)	(0.94 - 1.15)	(0.89 - 1.16)
At least once a week	0.83*	0.78	1.02	1.11
	(0.69 - 0.98)	(0.57 - 1.06)	(0.92 - 1.13)	(0.97 - 1.26)
**Region (Ref; North central)**				
North east	0.87	1.08	1.04	0.94
	(0.71 - 1.08)	(0.78 - 1.49)	(0.90 - 1.21)	(0.78 - 1.13)
North west	1.16	1.04	0.94	0.83
	(0.95 - 1.41)	(0.73 - 1.46)	(0.79 - 1.11)	(0.68 - 1.00)
South east	1.09	0.36***	1.73***	0.83
	(0.90 - 1.33)	(0.24 - 0.57)	(1.49 - 2.01)	(0.68 - 1.01)
South south	1.03	1.39	1.56***	1.62***
	(0.84 - 1.27)	(1.00 - 1.95)	(1.34 - 1.82)	(1.37 - 1.92)
South west	0.87	0.77	1.08	1.18
	(0.71 - 1.06)	(0.54 - 1.11)	(0.93 - 1.26)	(0.99 - 1.42)
Constant	1.36	0.19***	0.33***	0.20***
	(0.90 - 2.06)	(0.08 - 0.41)	(0.25 - 0.42)	(0.15 - 0.27)
Log pseudolikelihood	−8,944,000,000		−30,330,000,000	
Wald $\chi^{2}$	310.63		692.03	
Prob> $\chi^{2}$	0.00		0.00	
Observations	10,390		33,008	

Note: RRR; Relative Risk Ratio. 95% confidence interval (CI) in parentheses. Ref; Reference group. *** p < 0.001, ** p < 0.01, * p < 0.05.

The results show that men who have more sons are more likely (RRR = 1.47, 95% CI = 1.22–1.78) to prefer more boys rather than an equal number of boys and girls than men who have equal sons and daughters. Similarly, women who have more sons are more likely (RRR = 1.60, 95% CI = 1.45–1.77) to prefer more boys rather than an equal number of boys and girls compared to women who have equal sons and daughters. The regression results also indicate various demographic and cultural factors influencing parental sex preferences. However, age was not found to be a statistically significant predictor of sex preference in any of the models tested (see Table 2). This suggests that parental attitudes toward sex preference remain relatively stable across different age groups.

The probability of preferring more girls relative to an equal number of boys and girls for men who have more daughters are higher (RRR = 2.08, 95% CI = 1.52–2.84) compared to men who have the same number of sons and daughters. Likewise, the odds of preferring more girls relative to an equal number of boys and girls for women who have more daughters is higher (RRR = 1.65, 95% CI = 1.41–1.91) compared to men who have the same number of sons and daughters.

Women who have more sons are more likely (RRR = 1.15, 95% CI = 1.01–1.31) to prefer more girls rather than an equal number of boys and girls compared to women who have equal sons and daughters. Also, women who have no children have higher odds (RRR = 1.35, 95% CI = 1.18–1.55) of preferring more boys rather than an equal number of boys and girls than women who have equal sons and daughters. Furthermore, the probability of preferring more girls relative to an equal number of boys and girls for women who have no children is higher (RRR = 1.43, 95% CI = 1.19–1.71) than women who have the same number of sons and daughters.

### The relationship between culture and parental sex preferences among men and women in Nigeria

Table 3 shows the results of the relationship between culture and parental sex preferences among men and women in Nigeria. We found that Fulani, Hausa, Igala, and Kanuri/Beriberi men are more likely to prefer more boys rather than an equal number of boys and girls than Igbo men and this was phenomenal among Fulani men (RRR = 2.03, 95% CI = 1.37–3.01). However, Ibibio (RRR = 0.41, 95% CI = 0.27–0.63) and Tiv (RRR = 0.63, 95% CI = 0.41–0.95) men have a lower likelihood of preferring more boys rather than an equal number of boys and girls relative to Igbo men. Furthermore, the odds of preferring more girls relative to an equal number of boys and girls for Fulani, Hausa, Ijaw/Izon and Tiv men is high particularly for Hausa men (RRR = 3.58, 95% CI = 1.82–57.40) compared to Igbo men.

**Table 3:** Relationship between culture and parental sex preferences among men and women in Nigeria.

	Men 15–59		Women 15–49	
	Ref; B = G	Ref; B = G	Ref; B = G	Ref; B = G
	B > G	G > B	B > G	G > B
	RRR	RRR	RRR	RRR
Variables	(95%CI)	(95%CI)	(95%CI)	(95%CI)
**Ethnicity (Ref; Igbo)**				
Ekoi	–	–	0.84	2.07***
			(0.58 - 1.22)	(1.35 - 3.17)
Fulani	2.03***	2.48*	1.00	1.12
	(1.37 - 3.01)	(1.19 - 5.17)	(0.74 - 1.35)	(0.78 - 1.61)
Hausa	1.54*	3.58***	0.86	1.02
	(1.10 - 2.17)	(1.82 - 7.04)	(0.64 - 1.16)	(0.72 - 1.42)
Ibibio	0.41***	1.53	0.79	1.23
	(0.27 - 0.62)	(0.65 - 3.59)	(0.60 - 1.05)	(0.88 - 1.72)
Igala	1.99*	1.16	0.72	1.49*
	(1.18 - 3.37)	(0.46 - 2.93)	(0.45 - 1.17)	(1.03 - 2.17)
Ijaw/Izon	1.06	2.54**	0.97	1.49*
	(0.73 - 1.52)	(1.32 - 4.89)	(0.73 - 1.29)	(1.08 - 2.04)
Kanuri/Beriberi	1.66*	0.53	1.20	1.40
	(1.01 - 2.75)	(0.07 - 4.26)	(0.86 - 1.66)	(0.88 - 2.23)
Tiv	0.63*	2.26*	0.76	0.68
	(0.42 - 0.95)	(1.09 - 4.70)	(0.55 - 1.05)	(0.42 - 1.10)
Yoruba	1.33	1.73	0.69***	1.10
	(0.98 - 1.81)	(0.85 - 3.55)	(0.56 - 0.85)	(0.85 - 1.43)
Other	1.22	1.90*	0.91	1.17
	(0.93 - 1.60)	(1.08 - 3.35)	(0.72 - 1.14)	(0.88 - 1.55)
**Age (Ref; 15–19)**				
20-24	1.09	0.89	1.04	1.01
	(0.90 - 1.33)	(0.62 - 1.27)	(0.93 - 1.17)	(0.89 - 1.15)
25-29	1.03	0.94	1.01	0.99
	(0.84 - 1.28)	(0.63 - 1.39)	(0.90 - 1.14)	(0.86 - 1.14)
30-34	1.19	1.04	0.96	0.86
	(0.94 - 1.51)	(0.64 - 1.67)	(0.85 - 1.09)	(0.73 - 1.00)
35-39	1.09	1.38	0.94	1.02
	(0.85 - 1.40)	(0.83 - 2.30)	(0.83 - 1.08)	(0.85 - 1.22)
40-44	1.07	1.27	0.89	0.93
	(0.84 - 1.38)	(0.77 - 2.10)	(0.76 - 1.04)	(0.78 - 1.11)
45-49	1.26	1.14	0.90	0.95
	(0.95 - 1.67)	(0.66 - 1.98)	(0.77 - 1.04)	(0.79 - 1.15)
50-54	1.01	1.25		
	(0.74 - 1.37)	(0.71 - 2.22)		
55-59	1.02	0.75		
	(0.71 - 1.47)	(0.39 - 1.42)		
**Education (Ref; None)**				
Primary	1.00	0.99	1.26***	1.00
	(0.84 - 1.19)	(0.68 - 1.42)	(1.13 - 1.41)	(0.88 - 1.14)
Secondary	0.98	1.13	1.20**	0.98
	(0.83 - 1.16)	(0.81 - 1.58)	(1.06 - 1.36)	(0.84 - 1.15)
Higher	1.02	1.26	1.32***	1.04
	(0.83 - 1.25)	(0.85 - 1.87)	(1.13 - 1.55)	(0.84 - 1.28)
**Residence (Ref; Urban)**				
Rural	0.90	0.80	0.89*	1.01
	(0.79 - 1.03)	(0.63 - 1.03)	(0.80 - 0.98)	(0.89 - 1.14)
**Religion (Ref; Christian)**				
Islam	1.08	0.78	1.08	1.26*
	(0.91 - 1.29)	(0.54 - 1.14)	(0.95 - 1.22)	(1.04 - 1.53)
Traditionalist/Other	1.27	1.51	1.23	0.95
	(0.79 - 2.04)	(0.63 - 3.65)	(0.81 - 1.88)	(0.64 - 1.42)
**Wealth (Ref; Poorest)**				
Poorer	1.05	0.99	0.87*	0.86*
	(0.86 - 1.28)	(0.72 - 1.36)	(0.78 - 0.98)	(0.75 - 0.99)
Middle	1.03	0.95	0.81**	0.93
	(0.85 - 1.25)	(0.66 - 1.36)	(0.71 - 0.93)	(0.79 - 1.08)
Richer	0.88	1.02	0.77***	1.00
	(0.71 - 1.09)	(0.68 - 1.55)	(0.67 - 0.89)	(0.84 - 1.18)
Richest	0.72**	0.78	0.74***	0.93
	(0.56 - 0.91)	(0.49 - 1.25)	(0.63 - 0.86)	(0.77 - 1.12)
**Marital status (Ref; Never in union)**				
Married	0.92	0.90	1.06	0.85**
	(0.77 - 1.10)	(0.62 - 1.31)	(0.94 - 1.19)	(0.75 - 0.96)
Living with partner	1.07	1.10	1.26	0.93
	(0.63 - 1.82)	(0.55 - 2.19)	(1.00 - 1.60)	(0.74 - 1.18)
Widowed	0.77	0.69	1.24*	0.90
	(0.36 - 1.62)	(0.16 - 2.95)	(1.01 - 1.53)	(0.69 - 1.18)
Divorced	0.85	0.89	0.84	0.88
	(0.45 - 1.61)	(0.25 - 3.13)	(0.63 - 1.10)	(0.61 - 1.25)
Separated	1.01	1.15	1.14	0.92
	(0.51 - 2.03)	(0.39 - 3.37)	(0.88 - 1.48)	(0.67 - 1.26)
**Employment status (Ref; Unemployed)**				
Employed	1.02	1.09	1.09*	0.97
	(0.86 - 1.21)	(0.77 - 1.53)	(1.01 - 1.17)	(0.88 - 1.06)
**Frequency of listening to radio (Ref; Not at all)**				
Less than once a week	0.90	0.68*	0.85***	0.98
	(0.76 - 1.07)	(0.51 - 0.92)	(0.78 - 0.93)	(0.88 - 1.08)
At least once a week	0.91	0.76	0.83***	0.83***
	(0.77 - 1.07)	(0.56 - 1.03)	(0.76 - 0.91)	(0.75 - 0.92)
**Frequency of watching television (Ref; Not at all)**				
Less than once a week	1.02	0.93	1.05	1.03
	(0.86 - 1.21)	(0.70 - 1.24)	(0.95 - 1.16)	(0.90 - 1.17)
At least once a week	0.84*	0.80	1.02	1.12
	(0.71 - 1.00)	(0.59 - 1.08)	(0.92 - 1.13)	(0.99 - 1.28)
**Region (Ref; North central)**				
North east	0.75*	0.98	0.94	0.87
	(0.59 - 0.95)	(0.68 - 1.41)	(0.80 - 1.09)	(0.71 - 1.05)
North west	0.96	0.71	0.91	0.86
	(0.75 - 1.22)	(0.47 - 1.07)	(0.74 - 1.11)	(0.69 - 1.07)
South east	1.20	0.65	1.47**	0.86
	(0.88 - 1.64)	(0.32 - 1.31)	(1.14 - 1.91)	(0.61 - 1.20)
South south	1.08	1.35	1.47***	1.34**
	(0.85 - 1.36)	(0.92 - 1.99)	(1.23 - 1.75)	(1.10 - 1.63)
South west	0.79	0.86	1.24	1.12
	(0.59 - 1.04)	(0.52 - 1.42)	(1.00 - 1.54)	(0.87 - 1.43)
Constant	1.30	0.14***	0.50***	0.28***
	(0.88 - 1.94)	(0.06 - 0.30)	(0.37 - 0.68)	(0.20 - 0.40)
Log pseudolikelihood	−8,938,000,000		−30,470,000,000	
Wald $\chi^{2}$	333.75		507.29	
Prob> $\chi^{2}$	0.00		0.00	
Observations	10,390		33,008	

Note: RRR; Relative Risk Ratio. 95% confidence interval (CI) in parentheses. Ref; Reference group. *** p < 0.001, ** p < 0.01, * p < 0.05.

The results further revealed that Yoruba (RRR = 0.71, 95% CI = 0.57–0.88) women are less likely to prefer more boys relative to equal number of boys and girls than Igbo women. Also, the tendency of preferring more girls relative to an equal number of boys and girls for Ekoi, and Igala women are high, particularly for Ekoi women (RRR = 2.07, 95% CI = 1.35–3.17) compared to Igbo women. However, Tiv women (RRR = 0.64, 95% CI = 0.40–1.03) are less likely to prefer more girls relative to an equal number of boys and girls than Igbo women.

## Discussion

In this population-based study utilizing the most recent Nigeria Demographic and Health Survey (NDHS) data, we examined the relationship between family structure/cultural factors and parental sex preferences for children. The findings largely support our hypotheses. Consistent with H1, parents with more sons are significantly less likely to express a preference for additional boys, suggesting an implicit balancing behavior in family composition. H2 is also confirmed, as ethnic and religious variations in sex preferences are evident, with northern Nigerian groups exhibiting stronger son preference due to patrilineal inheritance customs. Finally, our results partially support H3, as maternal education and employment reduce the preference for sons, though the effect is stronger in urban areas compared to rural settings. These findings align with previous studies [28–33] while also highlighting new dynamics in parental sex preferences in Nigeria.

Our study revealed pronounced sex differences in these preferences, with a significant proportion of women (58.16%) expressing a preference for an equal number of boys and girls, compared to 40.67% of men. In contrast, a greater number of men (52.48%) preferred having more boys than women (26.2%), whilst more women (15.64%) preferred to have more girls compared to men (6.85%). These findings indicate that parental sex preferences in Nigeria are shaped by deeply embedded cultural and psychological factors. While some parents may prefer sons or daughters, this preference does not inherently imply gender-based disparities in treatment. Psychological theories suggest that parental sex preferences may be linked to social expectations, perceived economic advantages, or historical inheritance norms. Therefore, we expanded the scope beyond policy implications to provide a more holistic understanding of how cultural and psychological dimensions intersect with demographic trends [28–33].

Parental sex preferences in Nigeria exhibit notable regional variations, influenced by historical, economic, and cultural factors. In northern Nigeria, where patrilineal inheritance systems and extended family structures dominate, there is a pronounced preference for male children due to their role in preserving lineage and inheritance customs [34]. Sons are often perceived as economic assets, reinforcing the preference for boys [32]. In contrast, southwestern Nigeria, with relatively higher levels of female empowerment, urbanization, and economic participation of women, shows a more balanced preference [35]. Some communities demonstrate a growing acceptance of daughters as integral contributors to household stability, particularly as women achieve greater educational and professional opportunities [35,36]. Meanwhile, southeastern Nigeria, known for its strong entrepreneurial and trading culture, has traditionally favored sons for business continuity and inheritance [37]. However, recent socio-economic shifts, particularly in urban areas [35], suggest increasing gender parity in parental sex preferences as daughters play a more prominent role in supporting family enterprises and education-driven mobility.

These observed findings suggest that sex preference appears culturally profound, deeply rooted within societal norms, and traditional beliefs across various ethnic groups [38]. Nigeria as a country practice mainly a patrilineal system of family inheritance and as such a male child is a pride of contemporary Nigerian homes [39]. Hence it is not surprising that our findings are consistent with this trend and with prior literature that have reported son preference in Nigeria and other parts of the world [40–43]. Another plausible explanation for the widespread prevalence of son preference in Nigeria and other parts of Africa beyond the traditional belief that “sons belong to us and a daughter to someone else” [41] is the security that they believe sons bring. Research since the mid-1980s have shown that sons are preferred in African communities, including Nigeria, because they are perceived to contribute to preserving and maintaining family names, conferring social prestige and defense to parents, and provision of financial security at old age [32,41,44–46].

Although our study results do not align with those of Blau et al. [47], which reported a decline in son preference in the United States (US), recent reports indicate that this preference still exists in the US [48,49]. This suggests that despite the extensive awareness and legal provisions supporting gender equality and the desirability of both sexes in both developed and developing countries, research studies, including ours, continue to demonstrate a preference for sons.

Our study revealed that family structure is linked to parental sex preferences. Specifically, men with more sons are more likely to prefer additional boys compared to those with an equal number of sons and daughters, a trend that is statistically significant and aligns with broader reproductive behavior patterns observed in Nigeria. Similarly, women with more sons show a higher likelihood of preferring additional boys, while those with more daughters exhibit a greater preference for girls over an equal number of boys and girls. These findings are consistent with previous studies suggesting that fertility decisions in Nigeria are often made based on the existing composition of the family [28,50,51]. Research indicates that in some cultural contexts, women continue to bear children until they have an adequate number of sons to preserve their “marital status” and avoid divorce, resulting in shorter birth intervals and larger family sizes that extend into later childbearing ages [28]. This ongoing cycle significantly impacts the health of women and children, especially in marginalized communities, where healthcare access and family planning resources may be limited warranting a need for a strategic intervention.

Bongaarts’ study [52] on examining the trends for implementation for preferences for male offsprings among 41 countries in the world reported that cultural factors play a crucial role in shaping parental sex preferences. Our study supports this overall finding and extends it further by comprehensively assessing this association across the various ethnic groups in Nigeria. We found that men from Fulani, Hausa, Igala, Kanuri/Beriberi, and Yoruba ethnic groups were more likely to prefer more boys compared to Igbo men, with Fulani men showing the strongest preference. Conversely, men from Ibibio and Tiv ethnic groups were less likely to prefer boys, indicating significant cultural variations. For women, cultural influences were equally pronounced. Yoruba women were less likely to prefer more boys, while Ekoi women had a higher likelihood of preferring more girls. These findings reflect deep-rooted cultural norms shaping parental preferences across Nigeria’s diverse ethnic groups, as noted by prior studies attributing the persistence of son preference to patrilineal inheritance systems [52–54].

The findings of this study show that socioeconomic factors, particularly education and wealth status, significantly affect parental sex preferences. Parents with higher educational attainment and wealth showed a preference for a balanced number of sons and daughters. This trend aligns with global observations, where increased socioeconomic resources are associated with more egalitarian views on gender, suggesting that educational opportunities and economic empowerment could mitigate entrenched sex biases [55,56]. Additionally, our study highlights the regional variations in parental sex preferences. For instance, we observed a significant preference for sons in rural areas compared with urban areas. This finding is in agreement with a previous study that reported a significant son preference in both urban and rural areas of Enugu State, although balanced sex preferences were more prevalent in urban areas [30]. This suggests that urbanization and increased exposure to diverse cultural influences may lead to more equitable sex preferences. Nonetheless, such regional disparities emphasize the need for policymakers to tailor strategies to specific cultural and regional contexts to effectively address these preferences.

The preferences for male children have significant implications for family planning and public health. Families may continue to have children until the desired number of sons is achieved, potentially leading to larger family sizes. Large family sizes pose significant obstacles for rural families in providing proper nutrition, education, healthcare, housing, and support. In urban areas, high fertility rates exacerbate overcrowding and deteriorate living conditions. Given Nigeria’s status as one of the most populous countries globally, it is crucial to recognize that while population can be a valuable resource, unchecked growth may impede development and decline in living standards. Therefore, addressing the consequences of rapid population growth, influenced by sociocultural factors, such as the preference for male children, is essential for achieving the sustainable development goals (SDG) set out by the United Nations by 2030. In addition to the SDG, these dynamics can adversely affect maternal health, strain family resources, and affect child welfare. Public health interventions aimed at promoting sex balance within families should consider these dynamics to foster healthier family planning practices.

### Strengths and limitations

To the best of our knowledge, this is the first study conducted in the past decade to comprehensively assess the association between family structure and culture and parental sex preferences for children, extending the literature further by considering all ethnic groups and regions of Nigeria. The use of the most recent nationally representative data ensures that the study is highly generalizable to all regions of Nigeria, making it relevant for designing effective policies and interventions that will benefit the Nigerian people.

Despite these strengths, our study should be interpreted in light of the limitations. One key limitation is the potential endogeneity of family structure due to selective stopping. While we acknowledge that parents who prefer sons may continue childbearing until they achieve their desired family composition, we did not have access to suitable instrumental variables (IV) to formally address this issue. Also, we relied on self-reported data to assess our variables of interest, which are prone to social desirability bias and nondifferential misclassification. Should any of this happen, it would have biased our results towards the null. However, considering that we observed significant findings away from the null, our findings were less likely to have been greatly impacted by these biases. Additionally, the cross-sectional design of the NDHS limits our ability to draw causal inferences regarding the dynamics of parental sex preferences. Future studies employing longitudinal designs and mixed methods would provide deeper insights into the dynamics at play by exploring how these factors influence parental sex preferences and their long-term implications on demographic trends and public health as they change over time.

## Conclusion

The study has shown that parents (men and women) have different sex preferences for children, and this is linked to their respective family structure and their socio-demographic characteristics. Our study highlights the complex interplay between family structure, cultural background, socioeconomic status, and other demographic factors in shaping parental sex preferences in Nigeria. These insights are crucial for understanding gender dynamics and informing policies aimed at promoting sex equality and a balanced family structure. Such policies are essential for improving family planning practices and advancing towards the SDGs by 2030. With only six years remaining to achieve these goals, we call on all stakeholders to collaborate and take concerted efforts to make substantial progress.

## Supporting information

S1 File Appendix tables. (DOCX)
