# Supplementary material for: Parental preferences for sex of children in Nigeria: Cultural influences and family structure
Source: PLoS One. 2025 Jul 11;20(7):e0327474. doi: 10.1371/journal.pone.0327474 (PMC12250230; doi:10.1371/journal.pone.0327474)
Supplement: S1 File — (DOCX) [file pone.0327474.s001.docx]

**APPENDIX**

**Table 1A: VIF Results**

| **Variables** | **Men Sample - VIF** | **Women Sample - VIF** |
| --- | --- | --- |
| Age | 1.72 | 1.38 |
| Education level | 1.65 | 2.19 |
| Residence | 1.37 | 1.41 |
| Religion | 1.29 | 1.44 |
| Wealth | 2.29 | 2.28 |
| Marital status | 1.58 | 1.23 |
| Employment status | 1.22 | 1.18 |
| Frequency of listening to radio | 1.66 | 1.36 |
| Frequency of watching television | 2.01 | 1.98 |
| Region | 1.33 | 1.25 |

**Table A2: Relationship between family structure and parental sex preferences among men and women in Nigeria – Unadjusted model**

|  | **Men 15 – 59** | |  | **Women 15 – 49** | |
| --- | --- | --- | --- | --- | --- |
|  | B > G | G > B |  | B > G | G > B |
|  | (Ref; B = G) | (Ref; B = G) |  | (Ref; B = G) | (Ref; B = G) |
|  | RRR | RRR |  | RRR | RRR |
| Variables | (95%CI) | (95%CI) |  | (95%CI) | (95%CI) |
| **Family structure (Ref; Sons = Daughters)** |  |  |  |  |  |
| Sons > daughters | 1.50*** | 1.00 |  | 1.57*** | 1.14** |
|  | (1.25 - 1.81) | (0.70 - 1.42) |  | (1.43 - 1.74) | (1.00 - 1.29) |
| Daughters > sons | 1.05 | 2.00*** |  | 1.01 | 1.61*** |
|  | (0.89 - 1.24) | (1.47 - 2.72) |  | (0.91 - 1.13) | (1.39 - 1.87) |
| No children | 1.29*** | 1.42** |  | 1.28*** | 1.58*** |
|  | (1.09 - 1.53) | (1.03 - 1.94) |  | (1.14 - 1.43) | (1.38 - 1.80) |
| **Age (Ref; 15-19)** |  |  |  |  |  |
| 20-24 | 1.06 | 0.90 |  | 1.06 | 0.94 |
|  | (0.88 - 1.27) | (0.64 - 1.28) |  | (0.96 - 1.17) | (0.83 - 1.06) |
| 25-29 | 0.95 | 0.90 |  | 1.07 | 0.88** |
|  | (0.79 - 1.13) | (0.65 - 1.25) |  | (0.97 - 1.19) | (0.79 - 0.99) |
| 30-34 | 0.98 | 0.88 |  | 1.04 | 0.77*** |
|  | (0.82 - 1.17) | (0.62 - 1.25) |  | (0.94 - 1.16) | (0.68 - 0.87) |
| 35-39 | 0.91 | 1.14 |  | 1.03 | 0.90 |
|  | (0.76 - 1.08) | (0.83 - 1.55) |  | (0.93 - 1.15) | (0.78 - 1.04) |
| 40-44 | 0.88 | 0.99 |  | 0.97 | 0.82*** |
|  | (0.73 - 1.05) | (0.72 - 1.36) |  | (0.86 - 1.10) | (0.71 - 0.95) |
| 45-49 | 1.04 | 0.89 |  | 1.01 | 0.82*** |
|  | (0.85 - 1.27) | (0.60 - 1.31) |  | (0.89 - 1.14) | (0.71 - 0.95) |
| 50-54 | 0.86 | 0.98 |  |  |  |
|  | (0.67 - 1.09) | (0.65 - 1.49) |  |  |  |
| 55-59 | 0.91 | 0.58** |  |  |  |
|  | (0.69 - 1.20) | (0.35 - 0.97) |  |  |  |
| **Education (Ref; None)** |  |  |  |  |  |
| Primary | 0.82** | 0.79 |  | 1.35*** | 1.03 |
|  | (0.69 - 0.97) | (0.55 - 1.12) |  | (1.21 - 1.51) | (0.90 - 1.17) |
| Secondary | 0.71*** | 0.85 |  | 1.27*** | 1.12** |
|  | (0.61 - 0.82) | (0.65 - 1.11) |  | (1.16 - 1.39) | (1.01 - 1.24) |
| Higher | 0.66*** | 0.88 |  | 1.30*** | 1.12 |
|  | (0.55 - 0.79) | (0.64 - 1.22) |  | (1.15 - 1.48) | (0.96 - 1.31) |
| **Residence (Ref; Urban)** |  |  |  |  |  |
| Rural | 1.17*** | 1.15 |  | 0.87*** | 0.97 |
|  | (1.04 - 1.32) | (0.94 - 1.41) |  | (0.80 - 0.95) | (0.87 - 1.08) |
| **Wealth (Ref; Poorest)** |  |  |  |  |  |
| Poorer | 0.98 | 0.99 |  | 0.95 | 0.88* |
|  | (0.81 - 1.18) | (0.72 - 1.35) |  | (0.86 - 1.06) | (0.77 - 1.01) |
| Middle | 0.93 | 0.91 |  | 1.03 | 0.99 |
|  | (0.79 - 1.11) | (0.66 - 1.23) |  | (0.91 - 1.16) | (0.86 - 1.14) |
| Richer | 0.75*** | 0.88 |  | 1.06 | 1.12 |
|  | (0.63 - 0.90) | (0.64 - 1.22) |  | (0.95 - 1.18) | (0.97 - 1.29) |
| Richest | 0.56*** | 0.66*** |  | 1.06 | 1.08 |
|  | (0.47 - 0.67) | (0.49 - 0.90) |  | (0.94 - 1.18) | (0.94 - 1.24) |
| **Marital status (Ref; Never in union)** |  |  |  |  |  |
| Married | 0.94 | 0.97 |  | 0.94 | 0.79*** |
|  | (0.85 - 1.04) | (0.80 - 1.18) |  | (0.86 - 1.03) | (0.72 - 0.86) |
| Living with partner | 0.87 | 1.07 |  | 1.33** | 1.02 |
|  | (0.53 - 1.44) | (0.56 - 2.03) |  | (1.07 - 1.67) | (0.82 - 1.27) |
| Widowed | 0.71 | 0.82 |  | 1.21** | 0.80* |
|  | (0.35 - 1.44) | (0.21 - 3.31) |  | (1.00 - 1.46) | (0.62 - 1.03) |
| Divorced | 0.90 | 1.11 |  | 0.73** | 0.82 |
|  | (0.49 - 1.68) | (0.33 - 3.71) |  | (0.56 - 0.96) | (0.58 - 1.15) |
| Separated | 1.03 | 1.75 |  | 1.20 | 0.93 |
|  | (0.52 - 2.05) | (0.60 - 5.15) |  | (0.93 - 1.54) | (0.69 - 1.26) |
| **Employment status (Ref; Unemployed)** |  |  |  |  |  |
| Employed | 1.06 | 1.01 |  | 1.10*** | 0.91** |
|  | (0.92 - 1.22) | (0.75 - 1.34) |  | (1.03 - 1.18) | (0.85 - 0.99) |
| **Frequency of listening to radio (Ref; Not at all)** |  |  |  |  |  |
| Less than once a week | 0.83*** | 0.66*** |  | 0.95 | 1.01 |
|  | (0.73 - 0.95) | (0.52 - 0.83) |  | (0.87 - 1.03) | (0.92 - 1.11) |
| At least once a week | 0.71*** | 0.58*** |  | 0.92** | 0.88*** |
|  | (0.62 - 0.81) | (0.46 - 0.73) |  | (0.85 - 1.00) | (0.80 - 0.97) |
| **Frequency of watching television (Ref; Not at all)** |  |  |  |  |  |
| Less than once a week | 0.81*** | 0.75** |  | 1.11** | 1.07 |
|  | (0.71 - 0.92) | (0.59 - 0.93) |  | (1.02 - 1.22) | (0.95 - 1.21) |
| At least once a week | 0.61*** | 0.62*** |  | 1.06 | 1.16*** |
|  | (0.53 - 0.70) | (0.50 - 0.77) |  | (0.98 - 1.16) | (1.05 - 1.29) |
| **Region (Ref; North central)** |  |  |  |  |  |
| North east | 1.01 | 1.09 |  | 1.08 | 1.02 |
|  | (0.83 - 1.24) | (0.81 - 1.47) |  | (0.94 - 1.24) | (0.86 - 1.21) |
| North west | 1.32*** | 1.00 |  | 0.93 | 0.91 |
|  | (1.09 - 1.59) | (0.74 - 1.35) |  | (0.80 - 1.07) | (0.77 - 1.08) |
| South east | 0.86 | 0.34*** |  | 1.68*** | 0.73*** |
|  | (0.72 - 1.03) | (0.23 - 0.50) |  | (1.47 - 1.92) | (0.61 - 0.87) |
| South south | 0.80** | 1.19 |  | 1.49*** | 1.47*** |
|  | (0.65 - 0.97) | (0.89 - 1.60) |  | (1.30 - 1.71) | (1.26 - 1.72) |
| South west | 0.71*** | 0.68** |  | 1.07 | 1.15 |
|  | (0.58 - 0.87) | (0.49 - 0.95) |  | (0.93 - 1.23) | (0.95 - 1.39) |
|  |  |  |  |  |  |
| Observations | 10,390 | |  | 33,008 | |

Note: RRR; Relative Risk Ratio. 95% confidence interval (CI) in parentheses. Ref; Reference group.

*** p<0.01, ** p<0.05, * p<0.10

**Table A3. Relationship between culture and parental sex preferences among men and women in Nigeria – Unadjusted model**

|  | **Men 15 - 59** | |  | **Women 15 - 49** | |
| --- | --- | --- | --- | --- | --- |
|  | B > G | G > B |  | B > G | G > B |
|  | (Ref; B = G) | (Ref; B = G) |  | (Ref; B = G) | (Ref; B = G) |
|  | RRR | RRR |  | RRR | RRR |
| Variables | (95%CI) | (95%CI) |  | (95%CI) | (95%CI) |
| **Ethnicity (Ref; Igbo)** |  |  |  |  |  |
| Ekoi | - | - |  | 0.83 | 2.97*** |
|  |  |  |  | (0.61 - 1.14) | (2.08 - 4.25) |
| Fulani | 1.98*** | 2.49*** |  | 0.69*** | 1.32** |
|  | (1.54 - 2.55) | (1.56 - 3.97) |  | (0.58 - 0.81) | (1.06 - 1.65) |
| Hausa | 1.59*** | 3.19*** |  | 0.57*** | 1.15* |
|  | (1.36 - 1.85) | (2.27 - 4.49) |  | (0.50 - 0.65) | (0.98 - 1.35) |
| Ibibio | 0.38*** | 2.44** |  | 0.76** | 1.77*** |
|  | (0.26 - 0.56) | (1.18 - 5.02) |  | (0.62 - 0.94) | (1.39 - 2.26) |
| Igala | 1.94*** | 1.52 |  | 0.59** | 1.72*** |
|  | (1.22 - 3.10) | (0.68 - 3.41) |  | (0.36 - 0.95) | (1.28 - 2.29) |
| Ijaw/Izon | 0.95 | 4.32*** |  | 0.99 | 2.19*** |
|  | (0.71 - 1.26) | (2.69 - 6.95) |  | (0.82 - 1.21) | (1.73 - 2.77) |
| Kanuri/Beriberi | 1.49* | 0.61 |  | 0.81** | 1.64*** |
|  | (0.96 - 2.34) | (0.09 - 4.26) |  | (0.66 - 0.98) | (1.16 - 2.31) |
| Tiv | 0.65*** | 2.98*** |  | 0.55*** | 0.72* |
|  | (0.48 - 0.89) | (1.82 - 4.88) |  | (0.44 - 0.69) | (0.50 - 1.03) |
| Yoruba | 0.95 | 1.85*** |  | 0.60*** | 1.46*** |
|  | (0.80 - 1.14) | (1.22 - 2.80) |  | (0.53 - 0.68) | (1.21 - 1.77) |
| Other | 1.17** | 2.78*** |  | 0.72*** | 1.43*** |
|  | (1.01 - 1.35) | (2.00 - 3.86) |  | (0.65 - 0.81) | (1.24 - 1.66) |
| **Age (Ref; 15-19)** |  |  |  |  |  |
| 20-24 | 1.06 | 0.90 |  | 1.06 | 0.94 |
|  | (0.88 - 1.27) | (0.64 - 1.28) |  | (0.96 - 1.17) | (0.83 - 1.06) |
| 25-29 | 0.95 | 0.90 |  | 1.07 | 0.88** |
|  | (0.79 - 1.13) | (0.65 - 1.25) |  | (0.97 - 1.19) | (0.79 - 0.99) |
| 30-34 | 0.98 | 0.88 |  | 1.04 | 0.77*** |
|  | (0.82 - 1.17) | (0.62 - 1.25) |  | (0.94 - 1.16) | (0.68 - 0.87) |
| 35-39 | 0.91 | 1.14 |  | 1.03 | 0.90 |
|  | (0.76 - 1.08) | (0.83 - 1.55) |  | (0.93 - 1.15) | (0.78 - 1.04) |
| 40-44 | 0.88 | 0.99 |  | 0.97 | 0.82*** |
|  | (0.73 - 1.05) | (0.72 - 1.36) |  | (0.86 - 1.10) | (0.71 - 0.95) |
| 45-49 | 1.04 | 0.89 |  | 1.01 | 0.82*** |
|  | (0.85 - 1.27) | (0.60 - 1.31) |  | (0.89 - 1.14) | (0.71 - 0.95) |
| 50-54 | 0.86 | 0.98 |  |  |  |
|  | (0.67 - 1.09) | (0.65 - 1.49) |  |  |  |
| 55-59 | 0.91 | 0.58** |  |  |  |
|  | (0.69 - 1.20) | (0.35 - 0.97) |  |  |  |
| **Education (Ref; None)** |  |  |  |  |  |
| Primary | 0.82** | 0.79 |  | 1.35*** | 1.03 |
|  | (0.69 - 0.97) | (0.55 - 1.12) |  | (1.21 - 1.51) | (0.90 - 1.17) |
| Secondary | 0.71*** | 0.85 |  | 1.27*** | 1.12** |
|  | (0.61 - 0.82) | (0.65 - 1.11) |  | (1.16 - 1.39) | (1.01 - 1.24) |
| Higher | 0.66*** | 0.88 |  | 1.30*** | 1.12 |
|  | (0.55 - 0.79) | (0.64 - 1.22) |  | (1.15 - 1.48) | (0.96 - 1.31) |
| **Residence (Ref; Urban)** |  |  |  |  |  |
| Rural | 1.17*** | 1.15 |  | 0.87*** | 0.97 |
|  | (1.04 - 1.32) | (0.94 - 1.41) |  | (0.80 - 0.95) | (0.87 - 1.08) |
| **Wealth (Ref; Poorest)** |  |  |  |  |  |
| Poorer | 0.98 | 0.99 |  | 0.95 | 0.88* |
|  | (0.81 - 1.18) | (0.72 - 1.35) |  | (0.86 - 1.06) | (0.77 - 1.01) |
| Middle | 0.93 | 0.91 |  | 1.03 | 0.99 |
|  | (0.79 - 1.11) | (0.66 - 1.23) |  | (0.91 - 1.16) | (0.86 - 1.14) |
| Richer | 0.75*** | 0.88 |  | 1.06 | 1.12 |
|  | (0.63 - 0.90) | (0.64 - 1.22) |  | (0.95 - 1.18) | (0.97 - 1.29) |
| Richest | 0.56*** | 0.66*** |  | 1.06 | 1.08 |
|  | (0.47 - 0.67) | (0.49 - 0.90) |  | (0.94 - 1.18) | (0.94 - 1.24) |
| **Marital status (Ref; Never in union)** |  |  |  |  |  |
| Married | 0.94 | 0.97 |  | 0.94 | 0.79*** |
|  | (0.85 - 1.04) | (0.80 - 1.18) |  | (0.86 - 1.03) | (0.72 - 0.86) |
| Living with partner | 0.87 | 1.07 |  | 1.33** | 1.02 |
|  | (0.53 - 1.44) | (0.56 - 2.03) |  | (1.07 - 1.67) | (0.82 - 1.27) |
| Widowed | 0.71 | 0.82 |  | 1.21** | 0.80* |
|  | (0.35 - 1.44) | (0.21 - 3.31) |  | (1.00 - 1.46) | (0.62 - 1.03) |
| Divorced | 0.90 | 1.11 |  | 0.73** | 0.82 |
|  | (0.49 - 1.68) | (0.33 - 3.71) |  | (0.56 - 0.96) | (0.58 - 1.15) |
| Separated | 1.03 | 1.75 |  | 1.20 | 0.93 |
|  | (0.52 - 2.05) | (0.60 - 5.15) |  | (0.93 - 1.54) | (0.69 - 1.26) |
| **Employment status (Ref; Unemployed)** |  |  |  |  |  |
| Employed | 1.06 | 1.01 |  | 1.10*** | 0.91** |
|  | (0.92 - 1.22) | (0.75 - 1.34) |  | (1.03 - 1.18) | (0.85 - 0.99) |
| **Frequency of listening to radio (Ref; Not at all)** |  |  |  |  |  |
| Less than once a week | 0.83*** | 0.66*** |  | 0.95 | 1.01 |
|  | (0.73 - 0.95) | (0.52 - 0.83) |  | (0.87 - 1.03) | (0.92 - 1.11) |
| At least once a week | 0.71*** | 0.58*** |  | 0.92** | 0.88*** |
|  | (0.62 - 0.81) | (0.46 - 0.73) |  | (0.85 - 1.00) | (0.80 - 0.97) |
| **Frequency of watching television (Ref; Not at all)** |  |  |  |  |  |
| Less than once a week | 0.81*** | 0.75** |  | 1.11** | 1.07 |
|  | (0.71 - 0.92) | (0.59 - 0.93) |  | (1.02 - 1.22) | (0.95 - 1.21) |
| At least once a week | 0.61*** | 0.62*** |  | 1.06 | 1.16*** |
|  | (0.53 - 0.70) | (0.50 - 0.77) |  | (0.98 - 1.16) | (1.05 - 1.29) |
| **Region (Ref; North central)** |  |  |  |  |  |
| Northeast | 1.01 | 1.09 |  | 1.08 | 1.02 |
|  | (0.83 - 1.24) | (0.81 - 1.47) |  | (0.94 - 1.24) | (0.86 - 1.21) |
| North West | 1.32*** | 1.00 |  | 0.93 | 0.91 |
|  | (1.09 - 1.59) | (0.74 - 1.35) |  | (0.80 - 1.07) | (0.77 - 1.08) |
| South East | 0.86 | 0.34*** |  | 1.68*** | 0.73*** |
|  | (0.72 - 1.03) | (0.23 - 0.50) |  | (1.47 - 1.92) | (0.61 - 0.87) |
| South South | 0.80** | 1.19 |  | 1.49*** | 1.47*** |
|  | (0.65 - 0.97) | (0.89 - 1.60) |  | (1.30 - 1.71) | (1.26 - 1.72) |
| South West | 0.71*** | 0.68** |  | 1.07 | 1.15 |
|  | (0.58 - 0.87) | (0.49 - 0.95) |  | (0.93 - 1.23) | (0.95 - 1.39) |
| Observations | 10,390 | |  | 33,008 | |

Note: RRR; Relative Risk Ratio. 95% confidence interval (CI) in parentheses. Ref; Reference group.

*** p<0.01, ** p<0.05, * p<0.10
